# Factors influencing surgical outcomes in orbital trapdoor fracture

**DOI:** 10.1038/s41598-021-96920-5

**Published:** 2021-09-02

**Authors:** Yi-Hua Chen, Yueh-Ju Tsai, I-Shiang Tzeng

**Affiliations:** 1 Department of Ophthalmology, Linkou Chang Gung Memorial Hospital, No. 5, Fu-Hsing St., Guishan Dist., Taoyuan City, 33375 Taiwan; 2 College of Medicine, Chang Gung University, No.259, Wenhua 1st Rd., Guishan Dist., Taoyuan city, 33302 Taiwan; 3 Department of Research, Taipei Tzu Chi Hospital, No.289, Jiang-Kuo Rd., Xindian Dist., New Taipei City, 231 Taiwan

**Keywords:** Outcomes research, Paediatric research, Medical research, Risk factors

## Abstract

This study is aimed to determine the appropriate timing of performing surgical reduction on trapdoor fractures for bringing out better visual outcomes and the factors influencing surgical outcomes in this special subgroup of fracture victims. Data for 72 patients of orbital trapdoor fracture were analyzed retrospectively. Post-operative diplopia is the main posttreatment outcome of orbital bone fracture reduction. The receiver-operating characteristic (ROC) analysis indicated the cut-off point of surgical reduction timing at day 10 post-fracture. The rate of postoperative diplopia showed a significant difference between patients who underwent surgical reduction within 10 days and those who did after 10 days of injury (5.3% vs. 38.2%). Multivariate analysis revealed that preoperative infra-duction limitation (*p* = 0.02), muscle incarceration (*p* = 0.01), duration from injury to surgical reduction (*p* = 0.004), and postoperative supra-duction limitation (*p* = 0.004) were independent factors for poor surgical outcomes. In younger patients with head injury, the diagnosis of orbital trapdoor fracture should be kept in mind. Timely recognition and surgical reduction, especially within 10 days, can result in better visual outcomes without the occurrence of postoperative diplopia.

## Introduction

Orbital floor fractures are typically seen in facial blunt injuries that constitute 3–45% of all pediatric facial fractures^1^. Orbital trapdoor fracture is an anatomic subtype of orbital floor fracture, and is a commonly seen orbital floor fracture in children. It was first described by Soll and Poley^2^. Only 7% of adult patients with orbital blowout fractures showed a trapdoor-type pattern^3^. Owing to the greater elasticity in the bones of children, the hinged fractured bone tends to snap back to its original position and entraps the herniated tissues. Entrapment of extraocular muscles and/or other orbital tissues would result in limitations of ocular motility, diplopia, enophthalmos, pain, or severe oculo-cardiac reflex^4^. The most recommended surgical timing for repair is within 24–48 h, according to recent publications^5–9^. Permanent residual limitation of eye movements caused by ischemic damage of fibromuscular or muscular systems was re-emphasized by Giovanni Gerbino et al.^8^; however, they were not able to establish a clear cut-off period for the timing of the surgery without knowing the exact pathophysiology of the injured tissues. Children with trapdoor fractures often visit us via consultations from the ER or transfers from other hospitals. Delayed surgical repairs of trapdoor fractures may occur when the children present with white eyes or when their symptoms, such as nausea and vomiting following traumatic injuries, are misdiagnosed and managed as post-contusion syndrome.

## Methods

The present study is a retrospective, consecutive, comparative, and interventional case series of patients who received a diagnosis of orbital trapdoor fracture between September 1999 and September 2018 at a tertiary referral center in Northern Taiwan. This study followed the tenets of the Declaration of Helsinki and was approved by the Institutional Review Board (IRB) of Chang Gung Memorial Hospital in Taoyuan, Taiwan (IRB 201801432B0). A waiver of consent was granted because of the retrospective nature of the project and anonymous analysis of data by the IRB of Chang Gung Memorial Hospital in Taoyuan, Taiwan. All methods were performed in accordance with relevant guidelines and regulations. The diagnoses of orbital trapdoor fractures were confirmed by computed tomography (CT) of the orbit. The inclusion criteria were as follows: (1) patients who received a diagnosis of orbital trapdoor fracture at presentation or after image study, (2) those who presented to our emergency room or outpatient clinic seeking primary reduction, (3) those who were under the age of 25 years and had no history of diplopia or previous ocular trauma; and (4) those who were followed up for at least 3 months post-surgery. The exclusion criteria were as follows: (1) patients with concomitant ocular injuries with orbital fractures and (2) those with combined complex facial fractures.

Data on the demographic, clinical features at presentation, and medical history were obtained. Enophthalmos was detected if there was more than 2 mm of difference in the globe position by using Hertel exophthalmometer in sitting position. In children who could not cooperate, they were asked to lift their head and look up to let doctors evaluate them by visual inspection. To evaluate ocular motility disturbance, manual Hess screen testing and extraocular muscle movement (EOM) were performed at presentation and at 3-month follow-up after surgical treatment. We used Hess Area Ratio (HAR%), the percentage of square area of the affected side compared with the healthy side on the Hess chart, which was proven effective in predicting postoperative diplopia by Pier et al.^10^, to compare the improvement in ocular motility after surgical reduction. On orbital CT, the orbital fracture site was classified as per the classification in the study conducted by Takahashi et al.^3^: horizontally divided into lateral (A1) or medial (A2) to the infraorbital groove/canal, the inferomedial orbital strut (A3), and the medial orbital wall (A4); longitudinally divided into anterior third (S1), middle third (S2), and posterior third (S3). A missing inferior oblique muscle branch of the oculomotor nerve on CT imaging combined with findings of inferior oblique muscle underaction on preoperative Hess chart indicated the presence of incarceration of the inferior oblique muscle branch^11^.

After orbital trapdoor fracture was diagnosed, patients underwent surgical reduction under general anesthesia via conjunctival or sub-ciliary approaches. Artificial implants, such as porous polyethylene sheets (MEDPOR^Ⓡ^) or titanium mesh were placed after incarcerated tissues were totally released from the trapdoor sites and repositioned into the intra-orbital space.

The possible poor prognostic factors for postoperative diplopia were evaluated using Pearson’s chi-squared test, Fisher exact test, and Student’s t-test for univariate analyses and binary logistic regression for multivariate analysis. A receiver operating characteristic (ROC) curve was used to predict the appropriate surgical timing. The predictive performance levels of the combination variables for postoperative diplopia in C-statistics were also compared using the DeLong method. We used SPSS (version 23.0, SPSS Inc., Chicago, IL, USA) and MedCalc Statistical Software version 18.11.3 (MedCalc Software bvba, Ostend, Belgium) for statistical analysis. *p* ≤ 0.05 was considered statistically significant.

## Results

**Figure 1:**
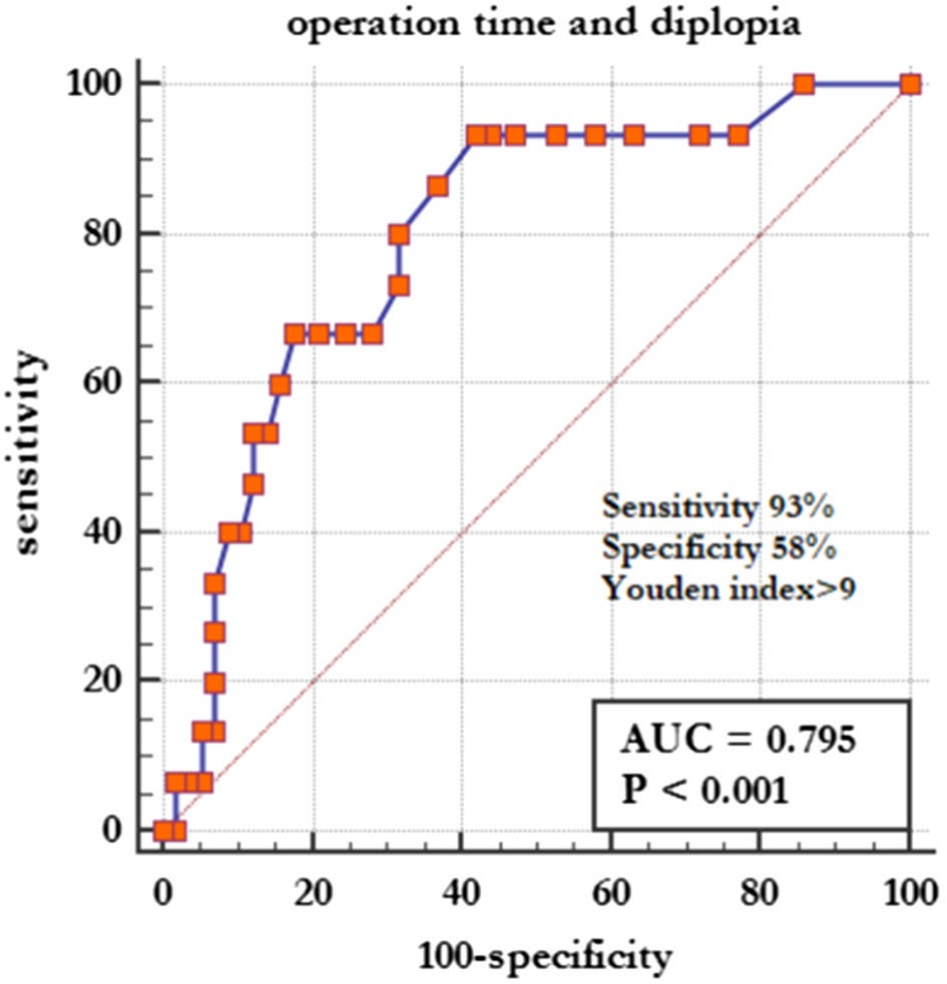
The receiving operator characteristic analysis indicated the cutoff point of surgical reduction timing in orbital trapdoor fracture. Maximum sensitivity and specificity were observed at day 10.

A total of 102 patients aged under 25 years had orbital floor or wall fractures between September 1999 and September 2018. Among them, 30 patients presented with blowout fractures without incarcerated tissues or muscles, and were therefore excluded from our study. Seventy-two patients had trapdoor fractures. Table 1 shows the patients’ characteristics, cause of injury, image results, and pre- and post-operative ocular features. Table 2 shows the prognostic factors for postoperative diplopia outcomes, and Table 3 implies the C-statistics analysis for the prediction of postoperative diplopia. Figure 1 resents the appropriate surgical timing.

**Table 1:** Data of patients’ characteristics, cause of injury, image results, and pre- and post-operative ocular features.

Demographics	Eye or patient no. (%)	Clinical features	Eye no. (%)
**Patient number**	72	**Pre-/post-operation**	
Eyes affected	72	Nausea and vomiting	35 (48.6%)/0
OD	41 (56.9%)	EOM limitation	67 (93.1%)/18 (25.0%)
OS	31 (43.1%)	Diplopia	68 (94.4%)/15 (20.8%)
**Mean age, years (± SD)**	14.0 ± 6.2	Bradycardia	5 (6.9%)/0
**Sex**		Enophthalmos	14 (19.4%)/1 (1.4%)
Male	47 (65.3%)	Hypoesthesia	9 (12.5%)/4 (5.6%)
Female	25 (34.7%)	Hess chart (HAR%) (± SD)	55.1 ± 22.0/93.7 ± 12.2
**Cause**		Supraduction limitation	66 (91.7%)/10 (13.9%)
Assault	22 (30.6%)	Infraduction limitation	39 (54.2%)/2 (2.8%)
Fall	15 (20.8%)	**Orbital CT**	
Sport	15 (20.8%)	**Fracture site**	
Traffic accident	15 (20.8%)	A1	1 (1.4%)
Other	5 (6.9%)	A2	66 (91.7%)
**Time**		A3	4 (5.6%)
From injury to surgical reduction (± SD)	17.1 ± 22.3	A4	1 (1.4%)
From injury to referral (± SD)	9.6 ± 19.2	**Anteroposterior fracture site**	
		S1	0
Incarcerated orbital tissue(± SD)	6.0 ± 3.0	S2	70 (97.2%)
Lesion IR (mm) (± SD)*	3.5 ± 0.8	S3	2 (2.8%)
Fellow IR (mm) (± SD)*	2.5 ± 0.6	**Trapdoor**	
Delta IR (mm) (± SD)*	1.1 ± 0.7	Without muscle	36 (50%)
Injury time to CT (days) (± SD)	6.6 ± 17.0	With muscle	36 (50%)

*IR* inferior rectus muscle, extraocular muscle movement, *SD* standard deviation, *CT* computed tomography, *EOM* extraocular muscle movement, *IR* inferior rectus muscle, *HAR* Hess area ratio.

*One eye with medial wall trapdoor fracture without floor involvement was excluded.

**Table 2:** Prognostic factors for postoperative diplopia outcomes.

Factors	No. of eyes	Post-op diplopia		Univariate		Multivariate	
		No diplopia	Diplopia	OR (95% CI)	*p* value	OR (95% CI)	*p* value
		No. of eyes (%)/Mean ± SD					
Cause: traffic accidents	15	8 (53.3)	7 (46.7)	5.5 (1.1–27)	0.034		
Pre-op infra-duction limitation	Y = 39	27(69.2)	12 (30.8)	4.4 (1.1–17.5)			
	N = 33	30 (90.9)	3 (9.1)	1	0.03	8.3 (1.4–101.8)	0.015
Trapdoor: muscle incarcerated	36	24 (66.7)	12 (33.3)	5.5 (1.4–21.6)			
Without muscle incarcerated	36	33 (91.7)	3 (8.3)	1	0.015	0.01 (0–0.3)	0.006
From injury to surgery (day)		13.7 ± 20.3	30.0 ± 25.5	1.0 (1.0–1.1)	0.029	1.2 (1.1–1.4)	0.004
From injury to referral (day)		7.4 ± 16.3	17.9 ± 26.4	1.0 (0.9–1.0)	0.092		
Post-op Supra-duction limitation	Y = 10	2 (20)	8 (80)	25.1 (1.7–5.04)		10.2 (2.0–71.6)	
	N = 62	55 (88.7)	7 (11.3)	1	< 0.001		0.004

OP operation, *SD* standard deviation, *OR* odds ratio, *CI* confidence interval.

**Table 3:** C-statistics for prediction of postoperative diplopia.

Variables	C-statistics (95% CI)	*p* value^a^
From injury to surgery > 9 days	0.756 (0.641–0.850)	–
Addition of post-op supra-duction limitation^a^	0.853 (0.705–0.926)	0.013
Further addition of muscle incarcerated^a^	0.906 (0.814–0.962)	0.048
Further addition of pre-op infra-duction limitation^a^	0.926 (0.839–0.974)	0.373

*OP* operation, *CI* confidence interval.

^a^Compared with the previous one.

### Patients’ characteristics

All 72 patients had unilateral involvement, and the right eye showed a higher percentage of trapdoor fractures (right eye, 41 of 72 eyes, 56.9%; left eye, 31 of 72 eyes, 43.1%). The mean age of our study group was 14.0 ± 6.2 years (range, 4–25 years). Boys were nearly two times (47 of 72 patients, 65.3%) more likely to have the condition than girls (25 of 72 patients, 34.7%); however, they did not exhibit substantial differences in their postoperative diplopia outcomes. The most common cause of trapdoor fracture was assault (22 of 72 eyes, 30.6%), followed by falls, sports, traffic accidents (15 of 72 eyes each, 20.8% each), and other causes (5 of 72 eyes, 6.9%). Traffic accidents were associated with poor postoperative diplopia outcomes (odds ratio [OR], 5.5; 95% confidence interval [CI] 1.1–27.0; *p* = 0.034).

### Clinical features at presentation

Common ocular complaints at the time of presentation to our hospital included limited extraocular muscle movement (EOM) (67 of 72 eyes, 93.1%) and diplopia (68 of 72 eyes, 94.4%). Less than half of the patients had nausea and vomiting (35 of 72 eyes, 48.6%). Enophthalmos was observed in 14 patients (19.4%). Only 9 of them had hypoesthesia (12.5%) and 5 had bradycardia (6.9%). Hess chart examination revealed a mean percentage of Hess area ratio (HAR%) of 55.1 ± 22.0% (19.2–100%). Sixty-six patients (93.1%) experienced supra-duction limitation, and thirty-nine patients (54.2%) had infra-duction limitation; however, poor postoperative diplopia outcomes (OR, 4.4; 95% CI 1.1–17.5; *p* = 0.03) were only observed in the infra-duction limitation group.

### CT examinations

Orbital CT was performed on all patients in this study. A2 (66 of 72 eyes, 91.7%) and S2 (70 of 72 eyes, 97.2%) trapdoor fractures occurred most frequently in trapdoor fractures of children and young adults. The presence or absence of postoperative diplopia did not differ in the distribution of orbital fracture sites. Muscle incarceration was observed in 36 patients (50%), which was considered to be a risk factor for postoperative diplopia outcomes (OR, 5.5; CI 1.4–21.6; *p* = 0.015). Soft tissue incarceration was found in other 36 patients (50%), which was not associated with postoperative diplopia outcomes. Incarceration of the inferior oblique muscle branch was confirmed in 12 patients (16.7%). Eight of them had concomitant muscle incarceration while the other 4 had soft tissue incarceration. The incarcerated orbital tissues were measured from the fracture site to the tip of the prolapsed tissues. The mean length of prolapsed tissue was 6.0 ± 3.0 mm (1.9–16.9 mm). We also measured the thickness of the inferior rectus muscle to determine the extent of swelling. The mean thickness was 3.5 ± 0.8 mm (2.1–6.1 mm) on the lesion side and 2.5 ± 0.6 mm (1.1–3.9 mm) on the fellow eye. The mean difference in the thickness of the inferior rectus muscle in the lesion and the fellow eyes was 1.1 ± 0.7 mm (0.1–2.7 mm). The inferior rectus muscle in eyes without muscle incarceration had a slightly larger thickness (3.7 ± 0.1 mm, 2.1–5.6 mm) than the eyes with muscle incarceration (3.4 ± 0.1 mm, 2.1–6.1 mm). However, no measured lengths or thicknesses showed statistical differences in postoperative diplopia outcomes. The mean duration from injury to CT examination was 6.6 ± 17.0 days (1–100 days). In addition, no correlation was found between the degree of swelling of inferior muscle and the days of CT image examination.

### Treatments

All patients underwent surgical reduction with Medpor^Ⓡ^ implantation, except three patients, wherein titanium mesh was used as the implant. The early detection of orbital trapdoor fractures is crucial. The mean duration from injury to surgical reduction was 17.1 ± 22.3 days (1–113 days), and it was significantly associated with poor postoperative diplopia outcomes (OR, 1.0; 95% CI 1.0–1.1; *p* = 0.029). In patients who sought medical help at the local medical department, the mean duration from injury to referral to medical center was 9.6 ± 19.2 days (0–105 days). None of the patients in our study received further corrective surgery of the extraocular muscles.

### Clinical features post-surgical treatment

Oculo-cardiac reflexes, such as bradycardia, nausea, and vomiting were completely absent immediately after surgical reduction. Enophthalmos was found in a 14-year-old boy (1 of 72 eyes, 1.4%) who had muscle incarcerated trapdoor fracture and underwent orbital floor reconstruction by Medpor^Ⓡ^ 25 days post-injury. He reported no diplopia and no limitation of EOM at the 3-month follow-up. At the 3-month follow-up, 4 patients complained of persistent hypoesthesia as in the preoperative stage (4 of 72 eyes, 5.6%). EOM limitation was seen in 18 patients (25%), but only 15 patients (20.8%) complained of diplopia. Postoperative Hess chart examination showed a mean percentage of HAR% of 93.7 ± 12.2% (83.3–100%). More patients had supra-duction limitation (10 of 72 patients, 13.9%) than infra-duction limitation (2 of 72 patients, 2.8%). Postoperative diplopia outcomes were significantly associated with supra-duction limitation (OR, 25.1; 95% CI 1.7–5.0; *p* < 0.001) and HAR% (OR, 0.7; 95% CI 0.6–0.9; *p* < 0.001).

### The appropriate surgical timing

The ROC analysis was used to indicate the cut-off point of surgical reduction timing in trapdoor orbital fractures. Maximum sensitivity and specificity were observed at day 10 with 93% sensitivity and 58% specificity. The area under the curve for the surgical reduction timing with trapdoor orbital fractures was 0.795 (*p* < 0.001). Thirteen of 34 patients (38.2%) who underwent surgical reduction later than 10 days had postoperative diplopia, but only 2 out of 38 patients (5.3%) reported to have diplopia after surgical reduction within 10 days.

### Multivariate analysis for postoperative diplopia outcomes

The significant poor prognostic factors (*p* < 0.05) for postoperative diplopia outcomes obtained from the univariate analysis (Table 2) were included in the multivariate analysis. Preoperative infra-duction limitation (OR, 8.3; 95% CI 1.4–101.8; *p* = 0.02), muscle incarceration (OR, 0.01; 95% CI 0–0.3; *p* = 0.01), duration from injury to surgical reduction (OR, 1.2; 95% CI 1.1–1.4; *p* = 0.004), and postoperative supra-duction limitation (OR, 10.2; 95% CI 2.0–71.6; *p* = 0.004) were important risk factors for postoperative diplopia compared with other significant factors of the univariate analysis.

### The C-statistics for prediction of postoperative diplopia

Table 3 shows the C-statistics indicating the predictive performance level for postoperative diplopia. The C-statistic was 0.756 for injury to surgery for more than 9 days, but when postoperative supra-duction and muscle incarceration were added to the regression model, significant stepwise improvements of the C-statistic (from 0.756 to 0.906 (*p* = 0.048)) were seen. However, adding preoperative infra-duction limitation did not further improve the predictive value for postoperative diplopia (*p* = 0.373).

## Discussion

In our study, assault was the most common cause of orbital trapdoor fractures in children and young adults, in age groups ranging from 6 to 25 years. This was compatible with the survey conducted by Yoon et al. in Korea, where assault accounted for 68.2% in 44 children^12^. Falls from heights occurred mostly in the preschool pediatric group who were diagnosed with orbital wall fractures in the study by Yang et al.^13^ As we noticed, 14 out of 15 children (93.3%) were under the age of 9 years in the group in which orbital trapdoor fractures were caused by falling from heights. Traffic accidents occurred most often in the older age group (10–25 years old).

Patients who present with nausea and vomiting after a traumatic episode, especially head injury, are usually evaluated as cases of concussion, which is a diagnosis of a mild traumatic brain injury. Symptoms of concussion also include changes in cognition, mood, visual-motor functioning, and balance^14^. However, nausea and vomiting also present as symptoms in orbital trapdoor fractures. Nausea and vomiting had been considered to have a predictive value of 71% for identifying orbital trapdoor fractures in the pediatric group by Jorden et al.^4^ Nearly half of the patients (48.6%) had symptoms in our study, but over 90% of our patients had EOM limitation and/or diplopia. If patients have a clear history of ocular blunt trauma or periorbital injury accompanied by symptoms of nausea, vomiting, and EOM limitation, further evaluation of orbital trapdoor fractures is suggested. Enophthalmos is not commonly seen in patients with isolated trapdoor fractures. Fourteen patients (19.4%) in this study had enophthalmos initially. We found out that their mean time from injury to referral was 26.1 ± 33.3 days which was much longer than average. We believed that prolonged local inflammation might result in enophthalmos.

We used HAR% as an objective parameter to evaluate ocular motility. It had proven effective in predicting diplopia in orbital blowout fractures by Pier et al.^10^ Our patients reported no diplopia with HAR% > 83.3%, and this was similar to that reported in other studies with HAR% > 85%^15^. Our postoperative HAR% showed a huge improvement from 55.1% ± 22.0% to 93.7% ± 12.2%, which was close to the final follow-up HAR% (92.9% ± 10.5%) examined by Yamanaka et al.^16^ We proved that it is an easy and reliable measurement of orbital trapdoor fracture patients after comparing it with patients’ subjective complaints and the results of previous studies.

Most of our cases had trapdoor fractures located at A2 (91.7%) and S2 (97.2%), where the bone of the orbital floor is thinner along the infraorbital groove/canal. Contusion or direct injury to the bundle of the infraorbital nerve is possible. In the pediatric group, due to thicker soft tissue over malar area, the hypoesthesia rate may be lower than in the adult group^17^. The hypoesthesia of infraorbital nerve is reversible. Only 4 patients reported persistent cheek numbness after 3 months of follow-up.

There were no correlations between postoperative diplopia outcomes and the distance from the fracture sites to the tip of incarcerated tissues and the swelling of the inferior rectus muscles. In addition, the delayed days of orbital CT filming did not show much or less swelling of the incarcerated inferior rectus muscles. We thought it was difficult to predict the surgical outcomes from the presentation of orbital CT. Takahashi et al. mentioned that in adult patients, the distance from the fracture sites to the tip of incarcerated tissues was shorter in the mid-late adulthood age group due to less displacement of bone when trapdoor fractures occurred^3^. However, in our study of patients aged under 25 years, we did not find a difference according to the age and the distance of prolapsed tissue.

Early surgical intervention ranging from within 24 h to 8 days in pediatric orbital trapdoor fractures has been emphasized in several studies^7,8,12,16,18^. In actual clinical practice, however, prompt surgery within several hours or days is difficult to achieve. Some children have already been “delayed” when they are referred^19^. We suggest having surgical reduction of inferior tissue entrapment within 10 days, post orbital trapdoor fractures. We believe it would bring out better outcomes on motility and result in less diplopia and would help in eradicating the necessity to have emergent surgical reduction in 48 h, which is difficult to achieve in rural areas. In the group that received surgical treatment within 10 days, the two patients who showed persistent diplopia were aged 5 and 10 years. They underwent surgery on day 10 and day 2, respectively. Inferior rectus muscle palsy was diagnosed and the muscle was thought to be injured during surgeries, which informed us the reduction of herniated tissue must be performed by pushing the trapdoor outward to prevent secondary injury to the inferior rectus muscle. Long term entrapment of the extraocular muscle causes ischemia and fibrotic changes, resulting in prolonged recovery of ocular mobility, or in permanent ocular restriction^4^. In the 15 postoperative diplopia cases, 12 were found to have muscle incarceration, whereas 3 of them had soft tissue incarceration. Soft tissue includes the sheath of the inferior rectus muscle, fibro-fatty tissues, and periosteum^20^. We found that not only muscle incarceration, but also prolonged soft tissue incarceration (26, 36, 51 days from injury to surgery) could lead to ocular restriction even after surgical reduction. Persistent mobility deficit due to fibrosis of the inferior rectus muscle and perimuscular tissue and their adhesion to the periosteum after reduction of orbital blowout fracture was noticed via magnetic resonance imaging by Okinaka et al.^21^.

Incarceration of the inferior oblique muscle branch happened in 12 patients (16.7%) aged between 6 and 21 years old, which was similar to the study conducted by Takahashi et al. (18.6%, range 5–19 years)^11^ but higher than Lee et al. (8.8%, range 6–24 years)^22^. Two of them who had concomitant muscle incarceration showed persisted diplopia after having surgery on day 2 and day 19. However, their postoperative Hess charts revealed underaction of inferior rectus muscle rather than inferior oblique muscle. While the other 10 patients reported no diplopia within 3 months after surgery. Also, no correlations between inferior oblique muscle branch incarceration and hypoesthesia, nausea and vomiting, and timing of surgery were found in this study. We suggest that the incarceration brings less effects on inferior oblique muscle branch than muscle and can be recovered after surgical reduction.

Multivariate logistic regression revealed that preoperative infra-duction limitation, muscle incarceration, postoperative supra-duction limitation, and the time from injury to surgical reduction were significant independent factors for poor postoperative diplopia outcomes. We found that the combination of independent covariates by C-statistics analysis further increased the predictive performance level of persistent diplopia in patients with trapdoor orbital fracture. Surgeons may add a fat-wrapped procedure to the inferior rectus muscle or postoperative anti-inflammation to decrease the fibrosis of the inferior rectus muscle and perimuscular tissue and their adhesion to the periosteum. Excursive exercise of extraocular muscle may prevent adhesion. However, there were several limitations to this study because of its retrospective nature. First, all patients included in this study were Asians; hence, the morphology was somewhat different from that of other ethnicities. Second, although we had 72 cases, a larger sample size is suggested to form a better picture of analysis. In addition, we followed up with the patients at 1 week, 1 month, and 3 months postoperatively; hence, we could not have an accurate day to check the pace of the recovery.

In conclusion, nausea and vomiting are commonly seen in orbital trapdoor fractures in children and young adults. When there is a traumatic history, it is necessary to check the EOM of the patient and perform an orbital CT. Early diagnosis and prompt surgical treatment within 10 days may salvage intact EOM and improve vision outcomes without diplopia.
